# Supplementary material for: Effects of the COVID-19 Pandemic on the Behavioural Tendencies of Cats and Dogs in Japan
Source: Animals (Basel). 2023 Jul 6;13(13):2217. doi: 10.3390/ani13132217 (PMC10340031; doi:10.3390/ani13132217)
Supplement: Supplementary file 1 [file animals-13-02217-s001.zip › animals-2445363-Supplementary_Materials.pdf]

Questionnaire 1: For cat owners

Survey of changes in the relationship between pets and people in the context of social change (cats)

* Indicates required question

1. If you are willing to participate in this survey, please check the "I agree" box and proceed. If you are under 20 years old or do not agree, please close your browser. *

   Check all that apply.

   - [ ] I agree

Tell us about yourself

2. Gender *

   Mark only one oval.

   - ( ) Female
   - ( ) Male
   - ( ) Other
   - ( ) I don't want to answer

3. Age (Please enter the age in digits only) *

   ____

4. Prefecture you live in *

Mark only one oval.

- Hokkaido
- Aomori
- Iwate
- Miyagi
- Akita
- Yamagata
- Fukushima
- Ibaraki
- Tochigi
- Gunma
- Saitama
- Chiba
- Tokyo
- Kanagawa
- Niigata
- Toyama
- Ishikawa
- Fukui
- Yamanashi
- Nagano
- Gifu
- Shizuoka
- Aichi
- Mie
- Shiga
- Kyoto
- Osaka
- Hyogo
- Nara
- Wakayama
- Tottori
- Shimane

- Okayama
- Hiroshima
- Yamaguchi
- Tokushima
- Kagawa
- Ehime
- Kochi
- Fukuoka
- Saga
- Nagasaki
- Kumamoto
- Oita
- Miyazaki
- Kagoshima
- Okinawa

5. Job style *

Mark only one oval.

- Full time job
- Part time job
- Student
- No job

6. Changes in their working/schooling conditions due to the COVID-19 pandemic *

Mark only one oval.

- stayed at home as before the state of emergency
- went from commuting to work or school to being home due to the state of emergency
- commuted to work or school but the number of times decreased due to the state of emergency
- went to work or school as before the state of emergenc

7. Current commuting status after lifting of emergency declaration *

Mark only one oval.

- ⬭ being at home
- ⬭ going to work or school as before the state of emergency
- ⬭ commuting to work or school, but less often than before the declaration

8. Human family members living with you *

Mark only one oval.

- ⬭ No family members living with me *Skip to question 16*
- ⬭ I have family members living with me *Skip to question 9*

Please tell us about the family members living with you. (First person)

9. Relationship with you (from your point of view)

Mark only one oval.

- ⬭ Spouse/Partner
- ⬭ Parent
- ⬭ Child
- ⬭ Relatives not listed above
- ⬭ Friend
- ⬭ Other: ______________________

10. Gender

Mark only one oval.

- ⬭ Female
- ⬭ Male
- ⬭ Other
- ⬭ I don't want to answer

11. Age (Please enter the age in digits only)

____

12. Job style

Mark only one oval.

- Full time job
- Part time job
- No job
- Students (College student / High school student / Junior high school student / Elementary school student)
- Kindergarten Child / Nursery Child
- Infants not attending preschool

13. Changes in their working/schooling conditions due to the COVID-19 pandemic

Mark only one oval.

- stayed at home as before the state of emergency
- went from commuting to work or school to being home due to the state of emergency
- commuted to work or school but the number of times decreased due to the state of emergency
- went to work or school as before the state of emergenc

14. Current commuting status after lifting of emergency declaration

Mark only one oval.

- being at home
- going to work or school as before the state of emergency
- commuting to work or school, but less often than before the declaration

15. Other family members living with you

Mark only one oval.

- ⬭ Not present *Skip to question 16*
- ⬭ There's more

If there were more than two family members (up to a sixth), respondents answered the same questions above for each family member.

Please tell us about the cat that is the subject of this response

16. Sex *

Mark only one oval.

- ⬭ Male
- ⬭ Female
- ⬭ Unknown

17. Age of the cat (if unknown, leave blank)

____

18. Spaying and neutering *

Mark only one oval.

- ⬭ Done
- ⬭ Not
- ⬭ Unknown

19. Age in months at which breeding started (if unknown, leave blank)

____

20. Origin *

Mark only one oval.

- Pet shop
- Breeder
- Shelter
- Unknown
- Other: ____________________

21. Breed (e.g., mongrel, American Shorthair; if unknown, leave blank)

____

22. Coat color (e.g., brown tabby and white; if unknown, leave blank)

____

23. Eye color (e.g. green; if unknown, leave blank)

____

24. Hair length *

Mark only one oval.

- Short
- Long
- Unknown
- Other: ____________________

25. Approximate weight (if unknown, leave blank)

____

26. If you have other cats, how many?

27. Please tell us if you have any other animals living with you in addition to the cat. (e.g., one dog)

Please answer the following questions about yourself

28. For each of the following five questions, choose the option that best describes your condition in the last two weeks. (There are six options. Please slide right to confirm) *

Mark only one oval per row.

	All the time	Most of the time	More than half of the time	Less than half of the time	Some of the time	At no time
**I have felt cheerful and in good spirits**	◯	◯	◯	◯	◯	◯
**I have felt calm and relaxed**	◯	◯	◯	◯	◯	◯
**I have felt active and vigorous**	◯	◯	◯	◯	◯	◯
**I woke up feeling fresh and rested**	◯	◯	◯	◯	◯	◯
**My daily life has been filled with things that interest me**	◯	◯	◯	◯	◯	◯

Please answer the following questions about your daily physical activity. Intense physical activity means any activity that feels physically demanding and causes you to breathe very unevenly. Moderate physical activity is defined as activity that is somewhat physically demanding and makes you a little out of breath. For the following questions, please consider only physical activity that lasts at least 10 minutes at a time.

29. In an average week, how many days do you engage in strong physical activity (carrying heavy loads, biking up hills, jogging, playing single tennis, etc.)?

Mark only one oval.

- 1
- 2
- 3
- 4
- 5
- 6
- 7
- 0

30. On days when you engage in strong physical activity, for how long do you typically engage in such activities in total per day? (approximate hours)

____

31. In an average week, how many days do you engage in moderate physical activity (carrying light loads, playing tags with children, slow swimming, tennis doubles, golf without a cart, etc.)? Please do not include walking or strolling in this list.

Mark only one oval.

- 1
- 2
- 3
- 4
- 5
- 6
- 7
- 0

32. On days when you engage in moderate physical activity, how long do you typically engage in such activity in total per day? (approximate hours)

____

33. In an average week, how many days do you engage in moderate physical activity (carrying light loads, playing tags with children, slow swimming, tennis doubles, golf without a cart, etc.)? Please do not include walking or strolling in this list.

Mark only one oval.

- 1
- 2
- 3
- 4
- 5
- 6
- 7
- 0

34. On such days, how long do you typically walk in total per day?

____

35. At what speed do you walk normally?

Mark only one oval.

- ( ) Quite fast, like breathing very unevenly
- ( ) A little breathless speed
- ( ) Slow and easy speed

36. This refers to the time you spend sitting or lying down every day (at work, studying, leisure time, etc.). Include all time spent at a desk, chatting with friends, reading, sitting, lying down, watching TV, etc. Do not include sleep time. On weekdays, how much time do you spend sitting or lying down in total per day?

____

37. During the holidays, how much time do you typically spend sitting or lying down in total per day?

____

For the following exercise questions, please select "yes" if applicable or "no" if not applicable.

38. Can climb up and down stairs

Mark only one oval.

- ( ) Yes
- ( ) No

39. No shortness of breath when climbing stairs

Mark only one oval.

- Yes
- No

40. Can jump up on both feet at the same time

Mark only one oval.

- Yes
- No

41. Can run about 20 steps

Mark only one oval.

- Yes
- No

42. Can overtake someone walking in front of you.

Mark only one oval.

- Yes
- No

43. Can walk for more than 30 min

Mark only one oval.

- Yes
- No

44. Can carry about objects weighing 5 kg

Mark only one oval.

- Yes
- No

45. Can lift objects weighing about 10 kg

Mark only one oval.

- Yes
- No

46. Can wake up a fallen bicycle

Mark only one oval.

- Yes
- No

47. Can open caps on plastic bottles

Mark only one oval.

- Yes
- No

48. Can touch the floor with fingertips while knees are extended

Mark only one oval.

- Yes
- No

49. Can put on socks, pants, skirts, etc. while standing without support

Mark only one oval.

- Yes
- No

50. Can get up from a chair without using hands

Mark only one oval.

- Yes
- No

51. Can stand on tiptoe without support

Mark only one oval.

- Yes
- No

Please indicate any changes in your involvement with the cat under the emergency declaration compared to before the COVID-19 outbreak led to calls for restraint in behavior.

52. Frequency of approaching the cat by themselves *

Mark only one oval.

- Increased
- Unchanged
- Decreased

53. Frequency of petting/touching the cat by themselves *

Mark only one oval.

- Increased
- Unchanged
- Decreased

54. Frequency of calling to the cat *

Mark only one oval.

- Increased
- Unchanged
- Decreased

55. Frequency of feeding (food) the cat *

Mark only one oval.

- Increased
- Unchanged
- Decreased

56. Frequency of giving treats to the cat *

Mark only one oval.

- Increased
- Unchanged
- Decreased

Please tell us about the behavior and condition of the cat that is the subject of this response.

The questions are divided into sections using simple language to help you identify feline behavior. Please answer all questions by selecting the appropriate option for each situation: what it looked like before the COVID-19 outbreak led to calls for behavioral restraint (before COVID-19), and what it looked like when it was restrained under the emergency declaration (after COVID-19). If you cannot answer a question (for example, you have never seen the cat in the situation described, it does not apply to my cat, etc.), please select "I have never encountered this situation" on the far right.
The Feline Behavioral Assessment & Research Questionnaire (Fe-BARQ) © James Serpell 2014

Please tell us about your family situation

57. What is the layout of your house? *

Mark only one oval.

- 1R、1K
- 2K、2DK、2LDK
- 3K、3DK、3LDK
- 4K、4DK、4LDK
- 5K、5DK、5LDK or more
- Don't know, don't want to answer

58. What is your annual household income (including taxes)? *

Mark only one oval.

- No income
- ~ less than 2 million yen
- 2-4 million yen
- 4-6 million yen
- 6-8 million yen
- 8-10 million yen
- 10-12 million yen
- 12-15 million yen
- 15-20 million yen
- 20 million yen or more
- Don't know, don't want to answer

59. What was the last school from which you graduated?

Mark only one oval.

- Junior high school
- High school
- Vocational school
- College of technology
- Junior college
- Four-year university
- Graduate school (including 6-year univ)
- Other

60. If you answered "other" above, please specify

____

61. A second survey will be scheduled after self-restraint and other conditions have completely improved. If you are willing to cooperate, please enter any 8-digit number (half-width characters) below and enter the same number for the second survey.

62. Please feel free to add any comments or suggestions you may have below. This is the last question. Thank you very much for your cooperation in this long survey!

This content is neither created nor endorsed by Google.

Google Forms

Questionnaire 2: For dog owners

Survey of changes in the relationship between pets and people in the context of social change (dogs)

* Indicates required question

1. If you are willing to participate in this survey, please check the "I agree" box and proceed. If you are under 20 years old or do not agree, please close your browser. *

Check all that apply.

☐ I agree

Tell us about yourself

2. Gender *

Mark only one oval.

◯ Female
◯ Male
◯ Other
◯ I don't want to answer

3. Age (Please enter the age in digits only) *

____

4. Prefecture you live in *

Mark only one oval.

- Hokkaido
- Aomori
- Iwate
- Miyagi
- Akita
- Yamagata
- Fukushima
- Ibaraki
- Tochigi
- Gunma
- Saitama
- Chiba
- Tokyo
- Kanagawa
- Niigata
- Toyama
- Ishikawa
- Fukui
- Yamanashi
- Nagano
- Gifu
- Shizuoka
- Aichi
- Mie
- Shiga
- Kyoto
- Osaka
- Hyogo
- Nara
- Wakayama
- Tottori
- Shimane

- Okayama
- Hiroshima
- Yamaguchi
- Tokushima
- Kagawa
- Ehime
- Kochi
- Fukuoka
- Saga
- Nagasaki
- Kumamoto
- Oita
- Miyazaki
- Kagoshima
- Okinawa

5. Job style *

Mark only one oval.

- Full time job
- Part time job
- Student
- No job

6. Changes in their working/schooling conditions due to the COVID-19 pandemic *

Mark only one oval.

- stayed at home as before the state of emergency
- went from commuting to work or school to being home due to the state of emergency
- commuted to work or school but the number of times decreased due to the state of emergency
- went to work or school as before the state of emergenc

7. Current commuting status after lifting of emergency declaration *

Mark only one oval.

- being at home
- going to work or school as before the state of emergency
- commuting to work or school, but less often than before the declaration

8. Human family members living with you *

Mark only one oval.

- No family members living with me *Skip to question 16*
- I have family members living with me *Skip to question 9*

Please tell us about the family members living with you. (First person)

9. Relationship with you (from your point of view)

Mark only one oval.

- Spouse/Partner
- Parent
- Child
- Relatives not listed above
- Friend
- Other: ____________________

10. Gender

Mark only one oval.

- Female
- Male
- Other
- I don't want to answer

11. Age (Please enter the age in digits only)

____

12. Job style

Mark only one oval.

- Full time job
- Part time job
- No job
- Students (College student / High school student / Junior high school student / Elementary school student)
- Kindergarten Child / Nursery Child
- Infants not attending preschool

13. Changes in their working/schooling conditions due to the COVID-19 pandemic

Mark only one oval.

- stayed at home as before the state of emergency
- went from commuting to work or school to being home due to the state of emergency
- commuted to work or school but the number of times decreased due to the state of emergency
- went to work or school as before the state of emergenc

14. Current commuting status after lifting of emergency declaration

Mark only one oval.

- being at home
- going to work or school as before the state of emergency
- commuting to work or school, but less often than before the declaration

15. Other family members living with you

Mark only one oval.

- ◯ Not present *Skip to question 16*
- ◯ There's more

If there were more than two family members (up to a sixth), respondents answered the same questions above for each family member.

Please tell us about the dog that is the subject of this response

16. Sex *

Mark only only one oval.

- ◯ Male
- ◯ Female
- ◯ Unknown

17. Age of the dog (if unknown, leave blank)

____

18. Spaying and neutering *

Mark only one oval.

- ◯ Done
- ◯ Not
- ◯ Unknown

19. Age in months at which breeding started (if unknown, leave blank)

____

20. Origin *

Mark only one oval.

- ⬭ Pet shop
- ⬭ Breeder
- ⬭ Shelter
- ⬭ Unknown
- ⬭ Other: ______________________

21. Breed (e.g. Golden Retriever. If it is a mix of two breeds, please write △△△×○○. If you have more than three breeds or an unknown mix breed (hybrid), please write "MIX". If unknown, leave blank)

____

22. Approximate weight (if unknown, leave blank)

____

23. If you have other dogs, how many?

____

24. Please tell us if you have any other animals living with you in addition to the dog. (e.g., one cat)

____

Please answer the following questions about yourself

25. For each of the following five questions, choose the option that best describes your condition in the last two weeks. (There are six options. Please slide right to confirm) *

Mark only one oval per row.

	All the time	Most of the time	More than half of the time	Less than half of the time	Some of the time	At no time
**I have felt cheerful and in good spirits**	⬭	⬭	⬭	⬭	⬭	⬭
**I have felt calm and relaxed**	⬭	⬭	⬭	⬭	⬭	⬭
**I have felt active and vigorous**	⬭	⬭	⬭	⬭	⬭	⬭
**I woke up feeling fresh and rested**	⬭	⬭	⬭	⬭	⬭	⬭
**My daily life has been filled with things that interest me**	⬭	⬭	⬭	⬭	⬭	⬭

Please answer the following questions about your daily physical activity. Intense physical activity means any activity that feels physically demanding and causes you to breathe very unevenly. Moderate physical activity is defined as activity that is somewhat physically demanding and makes you a little out of breath. For the following questions, please consider only physical activity that lasts at least 10 minutes at a time.

26. In an average week, how many days do you engage in strong physical activity (carrying heavy loads, biking up hills, jogging, playing single tennis, etc.)?

Mark only one oval.

- 1
- 2
- 3
- 4
- 5
- 6
- 7
- 0

27. On days when you engage in strong physical activity, for how long do you typically engage in such activities in total per day? (approximate hours)

____

28. In an average week, how many days do you engage in moderate physical activity (carrying light loads, playing tags with children, slow swimming, tennis doubles, golf without a cart, etc.)? Please do not include walking or strolling in this list.

Mark only one oval.

- 1
- 2
- 3
- 4
- 5
- 6
- 7
- 0

29. On days when you engage in moderate physical activity, how long do you typically engage in such activity in total per day? (approximate hours)

____

30. In an average week, how many days do you engage in moderate physical activity (carrying light loads, playing tags with children, slow swimming, tennis doubles, golf without a cart, etc.)? Please do not include walking or strolling in this list.

Mark only one oval.

- 1
- 2
- 3
- 4
- 5
- 6
- 7
- 0

31. On such days, how long do you typically walk in total per day?

____

32. At what speed do you walk normally?

Mark only one oval.

- Quite fast, like breathing very unevenly
- A little breathless speed
- Slow and easy speed

33. This refers to the time you spend sitting or lying down every day (at work, studying, leisure time, etc.). Include all time spent at a desk, chatting with friends, reading, sitting, lying down, watching TV, etc. Do not include sleep time. On weekdays, how much time do you spend sitting or lying down in total per day?

____

34. During the holidays, how much time do you typically spend sitting or lying down in total per day?

____

For the following exercise questions, please select "yes" if applicable or "no" if not applicable.

35. Can climb up and down stairs

Mark only one oval.

- Yes
- No

36. No shortness of breath when climbing stairs

Mark only one oval.

- Yes
- No

37. Can jump up on both feet at the same time

Mark only one oval.

- Yes
- No

38. Can run about 20 steps

Mark only one oval.

- Yes
- No

39. Can overtake someone walking in front of you.

Mark only one oval.

- Yes
- No

40. Can walk for more than 30 min

Mark only one oval.

- Yes
- No

41. Can carry objects weighing about 5 kg

Mark only one oval.

- Yes
- No

42. Can lift objects weighing about 10 kg

Mark only one oval.

- Yes
- No

43. Can wake up a fallen bicycle

Mark only one oval.

- Yes
- No

44. Can open caps on plastic bottles

Mark only one oval.

- Yes
- No

45. Can touch the floor with fingertips while knees are extended

Mark only one oval.

- Yes
- No

46. Can put on socks, pants, skirts, etc. while standing without support

Mark only one oval.

- Yes
- No

47. Can get up from a chair without using hands

Mark only one oval.

- Yes
- No

48. Can stand on tiptoe without support

Mark only one oval.

- Yes
- No

Please indicate any changes in your involvement with the dog under the emergency declaration compared to before the COVID-19 outbreak led to calls for restraint in behavior.

49. Frequency of approaching the dog by themselves *

Mark only one oval.

- Increased
- Unchanged
- Decreased

50. Frequency of petting/touching the dog by themselves *

Mark only one oval.

- Increased
- Unchanged
- Decreased

51. Frequency of calling to the dog *

Mark only one oval.

- Increased
- Unchanged
- Decreased

52. Frequency of feeding (food) the dog *

Mark only one oval.

- Increased
- Unchanged
- Decreased

53. Frequency of giving treats to the dog *

Mark only one oval.

- Increased
- Unchanged
- Decreased

54. Please choose how often you walk your canine from the list below. (There are seven options. Please slide right to confirm.) *

Mark only one oval per row.

	Twice a day	Once a day	2-4 times a week	Once a week	2 to 3 times a month	Almost never	Other
**Before COVID-19**	⬭	⬭	⬭	⬭	⬭	⬭	⬭
**After COVID-19**	⬭	⬭	⬭	⬭	⬭	⬭	⬭

55. Please select the length of your usual one walk *

Mark only one oval per row.

	More than 1 hour	More than 30 minutes but less than 1 hour	More than 15 minutes but less than 30 minutes	Less than 15 minutes
**Before COVID-19**	⬭	⬭	⬭	⬭
**After COVID-19**	⬭	⬭	⬭	⬭

Please tell us about the behavior and condition of the dog that is the subject of this response.

The questions are divided into sections using simple language to help you identify canine behavior. Please answer all questions by selecting the appropriate option for each situation: what it looked like before the COVID-19 outbreak led to calls for behavioral restraint (before COVID-19), and what it looked like when it was restrained under the emergency declaration (after COVID-19). If you cannot answer a question (for example, you have never seen the dog in the situation described, it does not apply to my dog, etc.), please select "I have never encountered this situation" on the far right.

The Canine Behavioral Assessment & Research Questionnaire (C-BARQ) ©James Serpell2005

Rearing situation

For each of the following questions, please select the number that applies to you before and after COVID-19.

56. Rearing Locations *

Mark only one oval per row.

	1. Indoor	2. Outdoor	3. Both indoor and outdoor
**Before COVID-19**	⬭	⬭	⬭
**After COVID-19**	⬭	⬭	⬭

57. Time spent in the same room as the dog per day. *

Mark only one oval per row.

	1. Less than 1 hour	2. More than 1 hour but less than 6 hours	3. More than 6 hours but less than 12 hours	4. More than 12 hours
**Before COVID-19**	⬭	⬭	⬭	⬭
**After COVID-19**	⬭	⬭	⬭	⬭

58. State in which the dog spends time when the owner is home *

Mark only one oval per row.

	1. Free to move	2. In a circle	3. Tie to a string
**Before COVID-19**	⬭	⬭	⬭
**After COVID-19**	⬭	⬭	⬭

59. State in which the dog spends time when the owner is away *

Mark only one oval per row.

	1. Free to move	2. In a circle	3. Crate (with door closed)	4. Tie to a string
**Before COVID-19**	⬭	⬭	⬭	⬭
**After COVID-19**	⬭	⬭	⬭	⬭

60. Whether the dog is allowed to accompany the owner when the owner is away from home (e.g., at work) and how the dog spends time there. *

Mark only one oval per row.

	1. Accompany and free there	2. Accompany, but tied up or in a cage there	3. Not accompany	4. Other
**Before COVID-19**	⬭	⬭	⬭	⬭
**After COVID-19**	⬭	⬭	⬭	⬭

61. Sleeping place at night (There are five options. Please slide right to confirm.) *

Mark only one oval per row.

	1. In a circle	2. They're on a string.	3. Inside the crate (with the door closed)	4. Sleeps in own bed or crate with freedom to move	5. In the same bed as the owner
**Before COVID-19**					
**After COVID-19**					

62. Approximately, how many hours per day do you usually leave your dog at home? Please choose one from the following. If it varies from day to day, please choose the most frequent option from the list below. (There are five options. Please slide right to confirm.) *

Mark only one oval per row.

	1. Less than 3 hours	2. More than 3 hours but less than 6 hours	3. More than 6 hours but less than 10 hours	4. More than 10 hours	5. Can't choose because of the variation from day to day.
**Before COVID-19**					
**After COVID-19**					

Please tell us about your family situation

63. What is the layout of your house? *

Mark only one oval.

- 1R、1K
- 2K、2DK、2LDK
- 3K、3DK、3LDK
- 4K、4DK、4LDK
- 5K、5DK、5LDK or more
- Don't know, don't want to answer

64. What is your annual household income (including taxes)? *

Mark only one oval.

- No income
- ~ less than 2 million yen
- 2-4 million yen
- 4-6 million yen
- 6-8 million yen
- 8-10 million yen
- 10-12 million yen
- 12-15 million yen
- 15-20 million yen
- 20 million yen or more
- Don't know, don't want to answer

65. What was the last school from which you graduated?

Mark only one oval.

- Junior high school
- High school
- Vocational school
- College of technology
- Junior college
- Four-year university
- Graduate school (including 6-year univ)
- Other

66. If you answered "other" above, please specify

____

67. A second survey will be scheduled after self-restraint and other conditions have completely improved. If you are willing to cooperate, please enter any 8-digit number (half-width characters) below and enter the same number for the second survey.

____

68. Please feel free to add any comments or suggestions you may have below. This is the last question. Thank you very much for your cooperation in this long survey!

This content is neither created nor endorsed by Google.

Table S1. Detailed attributions of participants

	Cat		Dog	
**Prefecture where the participants lived**				
	**No.**	**%**	**No.**	**%**
Hokkaido	28	4.58	27	4.68
Aomori	2	0.33	4	0.69
Iwate	5	0.82	1	0.17
Miyagi	12	1.96	7	1.21
Akita	2	0.33	3	0.52
Yamagata	3	0.49	4	0.69
Fukushima	6	0.98	2	0.35
Ibaragi	13	2.12	11	1.91
Tochigi	3	0.49	11	1.91
Gunma	2	0.33	3	0.52
Saitama	42	6.86	36	6.24
Chiba	35	5.72	31	5.37
Tokyo	167	27.29	151	26.17
Kanagawa	78	12.75	67	11.61
Niigata	6	0.98	6	1.04
Toyama	1	0.16	2	0.35
Ishikawa	3	0.49	4	0.69
Fukui	1	0.16	0	0
Yamanashi	5	0.82	1	0.17
Nagano	7	1.14	2	0.35
Gifu	4	0.65	8	1.39
Shizuoka	14	2.29	17	2.95
Aichi	35	5.72	27	4.68
Mie	4	0.65	6	1.04
Shiga	3	0.49	4	0.69
Kyoto	15	2.45	10	1.73
Ohsaka	27	4.41	26	4.51
Hyogo	17	2.78	23	3.99
Nara	2	0.33	6	1.04
Wakayama	0	0	1	0.17
Okayama	5	0.82	6	1.04
Tottori	1	0.16	1	0.17
Shimane	1	0.16	1	0.17

Hiroshima	8	1.31	11	1.91
Yamaguchi	4	0.65	2	0.35
Tokushima	2	0.33	0	0
Kagawa	5	0.82	5	0.87
Ehime	3	0.49	3	0.52
Kochi	2	0.33	1	0.17
Fukuoka	15	2.45	30	5.2
Saga	3	0.49	0	0
Nagasaki	1	0.16	4	0.69
Kumamoto	5	0.82	3	0.52
Ohita	5	0.82	3	0.52
Miyazaki	3	0.49	2	0.35
Kagoshima	4	0.65	3	0.52
Okinawa	3	0.49	1	0.17
Total	612	100	577	100

Job style of participant

	No.	%	No.	%
Full time	363	59.31	305	52.86
Part time	139	22.71	140	24.26
Student	11	1.80	9	1.56
No job	99	16.18	123	21.32
Total	612	100	577	100

Educational background of participants

	No.	%	No.	%
Graduate school (including 6-year univ)	44	7.19	41	7.11
Four-year university	233	38.07	213	36.92
Junior college	97	15.85	109	18.89
College of technology	13	2.12	17	2.95
Vocational school	95	15.52	82	14.21
High school	117	19.12	103	17.85
Junior high school	8	1.31	3	0.52
Others	3	0.49	3	0.52
NA	2	0.33	6	1.04
Total	612	100	577	100

Household income/year

	No.	%	No.	%
20 million yen or more	8	1.31	15	2.60
15-20 million yen	17	2.78	15	2.60
12-15 million yen	19	3.10	27	4.68
10-12 million yen	44	7.19	50	8.67
8-10 million yen	78	12.75	91	15.77
6-8 million yen	109	17.81	110	19.06
4-6 million yen	130	21.24	109	18.89
2-4 million yen	117	19.12	70	12.13
~ less than 2 million yen	27	4.41	14	2.43
No income	2	0.33	2	0.35
NA	61	9.97	74	12.82
Total	612	100	577	100

House layout

	No.	%	No.	%
5K、5DK、5LDK and more	86	14.05	107	18.54
4K、4DK、4LDK	123	20.10	153	26.52
3K、3DK、3LDK	173	28.27	171	29.64
2K、2DK、2LDK	162	26.47	99	17.16
1R、1K	50	8.17	23	3.99
NA	18	2.94	24	4.16
Total	612	100	577	100

Table S2. Attributions of cohabitants

	Cat		Dog	
**Age**				
	M	**SD**	***M***	***SD***
	46.92	21.67	46.92	21.67
**Gender**				
	**No.**	**%**	**No.**	**%**
Female	279	37.45	272	33.79
Male	463	62.15	522	64.84
Other/NA	3	0.40	11	1.37
Total	745	100	805	100
**Relationship**				
	**No.**	**%**	**No.**	**%**
Partner	308	41.40	364	45.16
Parents	206	27.69	193	23.95
Children	159	21.37	192	23.82
Other relatives	65	8.74	54	6.70
Friends	4	0.54	2	0.25
Other	2	0.27	1	0.12
Total	744	100	806	100
**Job sytle of cohabiting people**				
	**No.**	**%**	**No.**	**%**
Full time job	393	53.04	428	53.30
Part time job	71	9.58	60	7.47
No job	159	21.46	177	22.04
Student	95	12.82	123	15.32
Kindergarten/Nursery school	13	1.75	9	1.12
Infant/children at home	10	1.35	6	0.75
Total	741	100	803	100
**Changes in their working and schooling conditions due to the COVID-19 pandemic**				
	**No.**	**%**	**No.**	**%**

home / unchanged	189	26.00	220	27.64
office / unchanged	201	27.65	226	28.39
home / changed	183	25.17	208	26.13
office / changed	154	21.18	142	17.84
Total	727	100	796	100

Changes in their schooling conditions due to the COVID-19 pandemic of children (under 18)

	No.	%	No.	%
home / unchanged	12	13.79	7	9.46
office / unchanged	18	20.69	3	4.05
home / changed	53	60.92	56	75.68
office / changed	4	4.60	6	8.11
NA	0	0.00	2	2.70
Total	87	100	74	100

Age of children (under 18)

	M	SD	*M*	*SD*
	9.92	5.46	11.30	5.24

※Because of the missing values, the number of family members were different among tables.

Table S3. Attributions of cats

	M	SD	Effective data size
Age in years	7.04	4.91	582
Age in months at which breeding started	4.45	8.44	511
Body weight (kg)	4.63	1.33	596

Sex

	No.	%
Male	325	53.10
Female	287	46.90
Other/NA	0	0
Total	612	100

Neuterd

	No.	%
Done	582	95.10
No	29	4.74
NA	1	0.16
Total	612	100

Origin

	No.	%
Pet shop	70	11.44
Breeder	29	4.74
Shelter	158	25.82
Other	328	53.59
NA	27	4.41
Total	612	100

Breed

	No.	%
Mongrel	408	66.67
Russian blue	18	2.94
Scottish fold	18	2.94
American shorthair	23	3.76
Norwegian	9	1.47
Japanese	8	1.31

British shorthair	6	0.98
Maine coon	5	0.82
Ragdoll	5	0.82
American curl	4	0.65
Munchkin	4	0.65
Bengal	3	0.49
Minuet	3	0.49
Persian chinchilla	3	0.49
Exotic shorthair	2	0.33
Ragamuffin	2	0.33
Siberian	2	0.33
Singapura	2	0.33
Chartreux	1	0.16
Himalayan	1	0.16
Laperm	1	0.16
Oriental short hair	1	0.16
Somali	1	0.16
Tonkinese	1	0.16
NA	81	13.24
Total	612	100

Coat color

	No.	%
Brown tabby	82	13.40
Black and white	66	10.78
Brown tabby and white	60	9.80
Black	48	7.84
Calico	41	6.70
Red tabby	40	6.54
Gray	28	4.58
White	27	4.41
Tortoiseshell	21	3.43
Red and white	15	2.45
Other and NA	184	30.07
Total	612	100

Eye color

	No.	%
Green	205	33.50
Yellow	161	26.31
Gold	54	8.82
Yellow green	36	5.88
Blue	29	4.74
Other and NA	127	20.75
Total	612	100

Hair length

	No.	%
Short	516	84.31
Long	68	11.11
Middle	20	3.27
NA	8	1.31
Total	612	100

Cohabiting animals other than cats

	No.	%
Dog	50	8.17
Dog and other	8	1.31
Other	134	21.90
NA	420	68.63
Total	612	100

Table S4. Attributions of dogs

	M	SD	Effective data size
Age in years	7.55	4.35	561
Age in months at which breeding started	5.37	10.98	527
Body weight (kg)	8.48	6.49	568

Sex

	No.	%
Male	280	48.53
Female	297	51.47
Other/NA	0	0
Total	577	100

Neuterd

	No.	%
Done	467	80.94
No	110	19.06
NA	0	0.00
Total	577	100

Origin

	No.	%
Pet shop	264	45.75
Breeder	170	29.46
Shelter	74	12.82
Other	66	11.44
NA	3	0.52
Total	577	100

Breed

	No.	%
Mongrel	94	16.29
Shiba	80	13.86
Jack russell terrier	60	10.40
Toy poodle	60	10.40
Miniature dachshund	33	5.72
Chihuahua	19	3.29

Miniature schnauzer	19	3.29
Papillon	17	2.95
Welsh corgi	14	2.43
French bulldog	13	2.25
Pomeranian	13	2.25
Yorkshire errier	12	2.08
Golden retriever	11	1.91
Shih tzu	10	1.73
Maltese	10	1.73
Russian toy terrier	8	1.39
beagle	6	1.04
Labrador retriever	6	1.04
Long chihuahua	6	1.04
Cavalier	5	0.87
Shetland sheepdog	5	0.87
Pug	5	0.87
Border collie	5	0.87
Wire fox terrier	5	0.87
West highland white terrier	4	0.69
Miniature pinscher	4	0.69
American cocker spaniel	3	0.52
Italian greyhound	3	0.52
Boston terrier	3	0.52
German shepherd	2	0.35
Brussels griffon	2	0.35
Australian labradoodle	2	0.35
Pekingese	2	0.35
English golden	1	0.17
English bulldog	1	0.17
Australian shepherd	1	0.17
Kaninchen ducks	1	0.17
Cairn terrier	1	0.17
Samoyed	1	0.17
Siberian husky	1	0.17
Schnauzer	1	0.17
Smooth fox terrier	1	0.17
Chinese crested dog	1	0.17

Norfolk terrier	1	0.17
Bernese mountain dog	1	0.17
Basset hound	1	0.17
Bichon frize	1	0.17
Border terrier	1	0.17
Pomeranian	1	0.17
White shepherd	1	0.17
Miniature bull terrier	1	0.17
Long coat chihuahua	1	0.17
Weimaraner	1	0.17
Wire ducks	1	0.17
NA	15	2.60
Total	577	100

Cohabiting animals other than dogs

	No.	%
Cat	24	4.16
Cat and other	1	0.17
Other	17	2.95
NA	535	92.72
Total	577	100
